# Transcriptional Profiling of a Cross-Protective *Salmonella enterica* serovar Typhimurium UK-1 *dam* Mutant Identifies a Set of Genes More Transcriptionally Active Compared to Wild-Type, and Stably Transcribed across Biologically Relevant Microenvironments

**DOI:** 10.3390/pathogens3020417

**Published:** 2014-05-09

**Authors:** Claire B. Miller, Sebastian Aguilar Pierlé, Kelly A. Brayton, Jennine N. Ochoa, Devendra H. Shah, Kevin K. Lahmers

**Affiliations:** 1 Department of Veterinary Microbiology and Pathology, Washington State University, Pullman, WA 99164, USA; E-Mails: saguilar@vetmed.wsu.edu (S.A.P.); kbrayton@vetmed.wsu.edu (K.A.B.); jochoa@vetmed.wsu.edu (J.N.O.); dshah@vetmed.wsu.edu (D.H.S.); 2 Paul G. Allen School for Global Animal Health, Washington State University, Pullman, WA 99164, USA; 3 Department of Biomedical Sciences and Pathobiology, Virginia Tech, Blacksburg, VA 24061, USA

**Keywords:** *Salmonella* Typhimurium, DNA adenine methyltransferase, transcriptome, immunity, bacteriophage, fimbriae

## Abstract

Vaccination with *Salmonella enterica* serovar Typhimurium lacking DNA adenine methyltransferase confers cross-protective immunity against multiple *Salmonella* serotypes. The mechanistic basis is thought to be associated with the de-repression of genes that are tightly regulated when transiting from one microenvironment to another. This de-repression provides a potential means for the production of a more highly expressed and stable antigenic repertoire capable of inducing cross-protective immune responses. To identify genes encoding proteins that may contribute to cross-protective immunity, we used a *Salmonella* Typhimurium DNA adenine methyltransferase mutant strain (UK-1 *dam* mutant) derived from the parental UK-1 strain, and assessed the transcriptional profile of the UK-1 *dam* mutant and UK-1 strain grown under conditions that simulate the intestinal or endosomal microenvironments encountered during the infective process. As expected, the transcriptional profile of the UK-1 *dam* mutant identified a set of genes more transcriptionally active when compared directly to UK-1, and stably transcribed in biologically relevant culture conditions. Further, 22% of these genes were more highly transcribed in comparison to two other clinically-relevant *Salmonella* serovars. The strategy employed here helps to identify potentially conserved proteins produced by the UK-1 *dam* mutant that stimulate and/or modulate the development of cross-protective immune responses toward multiple *Salmonella* serotypes.

## 1. Introduction

Non-typhoidal *Salmonella* serotypes, including *Salmonella enterica* serovar Typhimurium and *Salmonella enterica* serovar Enteriditis, are food-borne pathogens, and important causes of bacterial enteric disease in both humans and livestock. Infection of livestock is a common source of transmission of *Salmonella* into the human food supply, and therefore interventions decreasing infection in livestock species have the potential to reduce the burden of human infection [1]. *Salmonella enterica* is an antigenically diverse species with approximately 2500 serotypes [2]. Multiple *Salmonella* serotypes can circulate concurrently on farms or production facilities resulting in antigenically distinct challenges to a single animal [3,4]. However exposure to one serotype does not result in significant cross-protection against heterologous serotypes [2]. In addition to serotype diversity, exposure of *Salmonella* to diverse microenvironments within the host serves as a cue to modulate its own gene transcription leading to differences in protein expression [5,6,7,8]. These changes in pathogen-expressed proteins in response to environmental cues result in skewed immune responses toward serotype-specific proteins which, in turn, contribute to the lack of cross-protection [9,10,11,12]. Therefore, strategies disrupting transcriptional shifts between microenvironments could result in sustained and increased production of conserved proteins providing the means for the adaptive immune system to induce efficient cross-protection against multiple serotypes.

Oral immunization of mice with a DNA adenine methyltransferase (Dam)-deficient *S.* Typhimurium (UK-1 *dam* mutant) confers significant protection against challenge with homologous *S.* Typhimurium, as well as heterologous challenge with serotypes Dublin and Enteritidis [13]. Subsequent studies showed similar results in other animal models, including cattle challenged with serotypes Dublin and Newport and chickens challenged with Enteritidis and strain O6, 14, 24 [14,15,16,17,18]. The underlying mechanism of protection has not been elucidated, but is thought to be due to de-repression of genes leading to an expanded antigenic repertoire [13]. It is known that mutation or removal of the *dam* gene from *Salmonella* causes de-repression of a number of genes under non-inducing culture conditions [19,20,21]. Genes in the SOS regulon, as well as fimbrial, conjugal transfer, bacteriophage and virulence-related genes have been reported to be up-regulated in *dam* mutant *Salmonella* Typhimurium strains [19,21]. Importantly, proteins corresponding to de-repressed genes, such as the StdA fimbrial subunit, have been detected within the outer membrane and culture supernatant, which are otherwise not present in wild-type (*wt*) *Salmonella* grown in identical conditions [19]. While these studies support the hypothesis that deficiency of Dam alters gene transcription in non-inducing culture conditions; the host environment is complex and the extent of these transcriptional alterations and their stability in host-like microenvironments is not known.

In this study, we address the hypothesis that the UK-1 *dam* mutant maintains higher, yet stable, gene transcription when compared with its isogenic *wt* strain, UK-1, in different host-like microenvironments. We employed a deep sequencing transcriptomics approach (RNA-Seq) to analyze gene expression by the UK-1 *dam* mutant in response to growth in laboratory media under high osmolarity (HSLB) to mimic the extracellular conditions in the intestinal lumen, and in acidic minimal media with low phosphate and low magnesium concentration (LPM) conditions found intracellularly within the *Salmonella* containing vacuole [22,23]. We identify stably expressed and highly transcribed genes in the UK-1 *dam* mutant in comparison to the parental strain when transitioning from an extracellular to an intracellular environmental condition. These findings were supported with comparison of two additional, clinically relevant *Salmonella* serotypes, Dublin and Newport, allowing us to identify a core gene repertoire whose transcription may play a role in inducing cross-protective immune response.

## 2. Results

### 2.1. UK-1 dam Mutant Produces Higher Levels of Transcription in Direct Comparison to UK-1 wt Parent Strain

**Figure 1:**
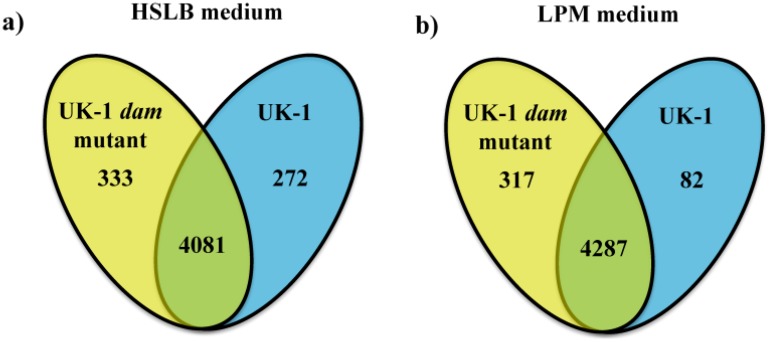
Number of genes more highly transcribed in direct comparison between the UK-1 *dam* mutant (yellow oval) and UK-1 *wt* parent strain (blue oval) in (**a**) High Salt Luria Burtani (HSLB) and (**b**) low phosphate and magnesium concentration (LPM) media. Genes with a 2-fold or higher cut off and a *p* < 0.05 were selected. The numbers in common in the Venn diagram represent genes transcript levels not significantly different between UK-1 *dam* mutant and UK-1.

Whole genome transcriptional activity in the UK-1 *dam* mutant and UK-1, its isogenic *wt* parent strain, were compared after growth in HSLB and LPM medium to identify differentially transcribed genes. Irrespective of the culture conditions used, the majority of the genes, 85% in HSLB and 91% in LPM, did not show significant transcriptional differences between the UK-1 *dam* mutant and the *wt* parent strain (Figure 1 and Supplementary Table 1). In the UK-1 *dam* mutant, 333 genes were identified with greater than 2-fold change and *p* < 0.05 in comparison to the *wt* parent strain when grown in HSLB. A similar comparison in the *wt* parent strain identified 272 genes in comparison to the UK-1 *dam* mutant (Figure 1a). When grown in LPM medium, 317 genes were highly transcribed in the UK-1 *dam* mutant whereas only 82 genes were highly transcribed in the *wt* parent strain (Figure 1b). In both culture conditions the UK-1 *dam* mutant transcribed genes more highly than the *wt* parent strain: 18% more genes in HSLB medium and 74% more genes in LPM medium. The proportion of highly transcribed genes was significantly different between the UK-1 *dam* mutant and the *wt* parent strain in both culture conditions (Fisher’s Exact test, *p*-value < 0.0001). The increased relative transcription observed in the UK-1 *dam* mutant in both culture conditions in comparison to the *wt* parent strain indicates that de-repression is consistent across different host-like microenvironments.

**Figure 2:**
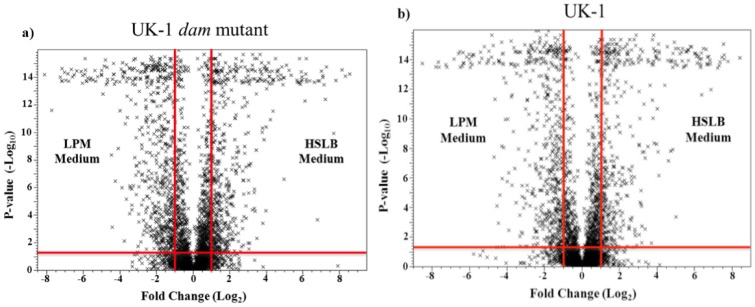
Volcano plots of intra-strain transcription comparing (**a**) UK-1 *dam* mutant grown in HSLB and LPM media; and (**b**) UK-1 *wt* parent strain grown in HSLB and LPM media. The x-axis is the log_2_ transformed fold change. The y-axis represents the -log_10_ transformed *p*-value. The vertical red lines denote a 2-fold change in transcription while the horizontal red line represents statistical significance at *p* < 0.05. The genes represented by X’s contained within the two vertical red lines, regardless of the *p*-value statistical significance cutoff, are considered stably transcribed genes in response to different microenvironments.

### 2.2. UK-1 dam Mutant and UK-1 wt Parent Strain Show Comparable Stable Transcription in Response to Growth in Differing Microenvironments

An intra-strain comparison was performed to identify stably transcribed genes with less than 2-fold change in response to environmental change. Transcription after growth in HSLB medium was compared to transcription after growth in LPM medium within a single strain. This gene set was then used to isolate genes that show high relative transcription in the two strains for further analysis. In the UK-1 *dam* mutant, a total of 2816 genes were stably transcribed, while 2833 genes were stably transcribed in the *wt* parent strain, representing approximately 60% of genes in both strains. The distribution of stably, as well as significantly transcribed genes (>2 fold with *p* < 0.05), was comparable between UK-1 *dam* mutant and the *wt* parent strain in both culture conditions, with no statistically significant differences (Figure 2a,b).

### 2.3. Identification of Genes More Highly Transcribed by the UK-1 dam Mutant and UK-1 wt Parent Strain Regardless of Culture Condition

**Figure 3:**
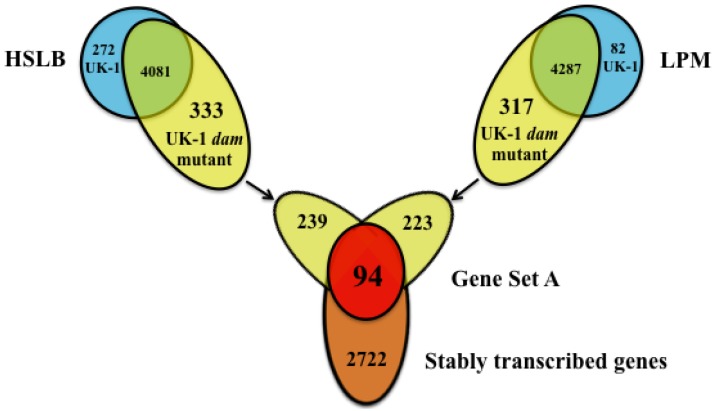
Analysis used for inclusion in Gene Set A for the UK-1 *dam* mutant. Selected genes were >2-fold more transcriptionally active in both culture conditions when directly compared to UK-1 (upper Venn diagrams), and between 2 and -2-fold in an intra-strain comparison (orange circle). Ninety-four genes were found to fit both criteria in the UK-1 *dam* mutant (red circle. (*p* < 0.05).

Ninety-four genes (Table 1), referred to as Gene Set A, were identified that (i) had significantly higher relative transcription by the UK-1 *dam* mutant in both culture conditions when compared with the *wt* parent strain; and (ii) were stably transcribed between HSLB and LPM media in the intra-strain comparison (Figure 3). For further analysis, we focused on this set of genes because their transcription is always “on” in the mutant regardless of environmental stimuli, and coupled with high relative transcription compared to the *wt* parent strain, could represent an important set of stably expressed target proteins promoting the development of cross-protection elicited by the UK-1 *dam* mutant. The average fold change of the 94 genes in HSLB medium was 13.29 (median 7.19), and ranged from 2.08-fold to 350-fold. Of all the 94 genes, the most highly transcribed were the two members of the *std* fimbrial operon, STMUK_3014 and STMUK_3015 with significant fold changes of 350 and 267 when UK-1 *dam* mutant was grown in HSLB medium. The average fold change in LPM medium was 13.19 (median 9.57), with a range of 2.02-fold to 119-fold. Genes in the *std* operon were also the most highly transcribed genes in LPM medium by the UK-1 *dam* mutant at 93-fold and 119-fold, respectively. These genes were grouped into 10 general functional categories based on the annotated *wt* parent strain sequence (Genbank accessions CP002614.1 and CP002615.1). Bacteriophage genes were most highly represented in Gene Set A with 48 genes (51%), all corresponding to the ST64B bacteriophage. The remaining genes fell into the categories including fimbriae (2%), SOS regulon (18%), replication (1%), metabolism (2%), transport (2%), translation (9%), virulence (1%), hypothetical proteins (11%), and genes contained on the plasmid (3%) (Table 1). A signal peptide analysis was applied on the predicted proteins of Gene Set A. Four of the 94 genes were predicted to have cell surface trafficking [24]. These genes included: the fimbrial protein STMUK_3015, a ST64B bacteriophage portal gene STMUK_2003, a Lex-A regulated gene of unknown function *ydjM*, and STMUK_1011, a bacteriophage attachment and invasion protein. In order to gain insight into probable function of identified hypothetical proteins, a BLAST analysis was performed. STMUK_0985 had 100% nucleotide sequence identity with a gene encoding a DNA damage inducible protein whereas its neighbor STMUK_0986 had 100% identity with a gene encoding a Gifsy-2 bacteriophage protein. STMUK_1849 and STMUK_2565 had 100% and 96% identity respectively with phage-associated genes, whereas STMUK_2239, STMUK_2240, *rtcB* and *yeeA* did not reveal any similarities with genes of known functions. STMUK_2656 did not have a clear similarity with any one gene as it had very high identity with multiple genes of unrelated function.

**Table 1:** Gene Set A.

Functional Category	Gene ID	Fold Change of UK-1 *dam* Mutant ^a^				Gene Product
		*vs*. UK-1 in HSLB	*vs*. UK-1 in LPM	*vs*. Dublin in HSLB	*vs*. Newport in HSLB	
Fimbriae	STMUK_3014 **^b^**	350.44	93.28	350.55	859.27	Hypothetical protein; *std* operon
	STMUK_3015	267.40	119.79	239.21	NP **^c^**	Putative outer membrane protein; *std* operon
Bacteriophage	STMUK_1981	6.81	4.53	15.96	NP	Hypothetical bacteriophage protein
	STMUK_1982	6.90	10.08	17.47	NP	Putative phage tail-like protein
	STMUK_1983	8.27	11.74	15.83	NP	Hypothetical bacteriophage protein
	STMUK_1984	8.06	9.40	15.30	NP	Similar to tail protein
	STMUK_1985	6.32	7.46	25.19	NP	Similar to tail protein
	STMUK_1986	4.91	10.49	18.11	NP	Similar to phage protein
	STMUK_1987	9.94	7.56	35.25	NP	Putative bacteriophage protein
	STMUK_1988	8.74	8.21	21.68	NP	Putative bacteriophage protein
	STMUK_1989	7.30	10.28	24.59	NP	Putative bacteriophage protein
	STMUK_1990	7.13	13.77	29.52	NP	Putative bacteriophage protein
	STMUK_1991	7.04	13.51	32.31	NP	Hypothetical bacteriophage protein
	STMUK_1992	8.08	18.23	23.22	NP	Putative tail tube protein
	STMUK_1993	7.41	22.23	80.27	NP	Putative bacteriophage protein
	STMUK_1994	7.28	21.15	69.99	NP	Hypothetical bacteriophage protein
	STMUK_1995	8.96	30.02	76.36	NP	Hypothetical bacteriophage protein
	STMUK_1996	9.84	26.91	150.38	NP	Hypothetical bacteriophage protein
	STMUK_1997	7.64	23.75	98.95	NP	Hypothetical bacteriophage protein
	STMUK_1998	8.35	21.73	43.65	NP	Hypothetical bacteriophage protein
	STMUK_1999	7.27	24.96	66.19	NP	Hypothetical bacteriophage protein
	STMUK_2000	7.11	24.09	189.35	NP	Putative bacteriophage protein
	STMUK_2001	7.97	25.17	311.71	NP	Putative bacteriophage protein
	STMUK_2002	9.29	19.64	52.79	NP	Putative bacteriophage protein
	STMUK_2003	6.66	22.35	68.68	NP	Hypothetical bacteriophage protein
	STMUK_2004	7.86	20.07	30.54	NP	Putative bacteriophage protein
	STMUK_2005	10.31	23.71	28.43	NP	Hypothetical bacteriophage protein
	STMUK_2006	8.28	16.09	80.27	NP	Hypothetical bacteriophage protein
	STMUK_2007	8.38	18.22	404.88	352.87	Putative bacteriophage protein
	STMUK_2008	9.24	24.28	NP	NP	Hypothetical bacteriophage protein
	STMUK_2009	3.68	5.56	NP	NP	Hypothetical bacteriophage protein
	STMUK_2010	8.16	8.61	NP	NP	Hypothetical bacteriophage protein
	STMUK_2011	7.18	7.15	9.98	NP	Putative cytoplasmic protein
	STMUK_2012	7.72	8.67	5.83	NP	Hypothetical bacteriophage protein
	STMUK_2013	12.83	9.73	7.24	NP	Hypothetical bacteriophage protein
	STMUK_2014	10.00	16.93	NP	NP	Putative bacteriophage protein
	STMUK_2015	10.12	21.50	NP	NP	Hypothetical bacteriophage protein
	STMUK_2016	7.86	31.44	6.02	NP	Hypothetical bacteriophage protein
	STMUK_2017	9.07	17.72	NP	NP	Hypothetical bacteriophage protein
	STMUK_2018	9.06	13.96	4.03	NP	Hypothetical bacteriophage protein
	STMUK_2019	7.35	12.62	4.26	NP	Hypothetical bacteriophage protein
	STMUK_2021	13.14	26.39	3.18	NP	Hypothetical bacteriophage protein
	STMUK_2022	11.69	20.75	3.46	NP	Hypothetical bacteriophage protein
	STMUK_2023	12.09	24.14	2.81	NP	Similar to phage protein
	STMUK_2024	8.37	12.71	NP	NP	Hypothetical bacteriophage protein
	STMUK_2025	9.37	10.70	NP	NP	Hypothetical bacteriophage protein
	STMUK_2026	10.27	9.84	NP	NP	Hypothetical bacteriophage protein
	STMUK_2027	5.98	7.97	7.79	NP	Putative bacteriophage protein
	STMUK_2028	7.20	12.42	5.09	NP	Hypothetical bacteriophage protein
	STMUK_2029	4.88	4.19	2.62	NP	Similar to phage integrase
SOS regulon	STMUK_1276	3.92	5.28	−1.18	2.93	Nucleotide excision repair endonuclease
	*dinF*	4.50	4.68	2.42	2.04	DNA-damage-inducible SOS response protein
	*dinP*	4.76	5.07	2.67	2.13	DNA polymerase IV
	*lexA*	4.15	3.90	2.34	2.31	LexA repressor
	*polB*	2.66	2.73	2.17	NP	DNA polymerase II
	*recA*	4.86	4.86	3.42	3.18	Recombinase A
	*recN*	10.81	10.62	5.80	4.34	Recombination and repair protein
	*ruvA*	2.78	2.57	2.43	2.30	Holliday junction DNA helicase RuvA
	*ruvB*	2.28	2.11	2.49	2.04	Holliday junction DNA helicase
	*sulA*	6.17	11.44	2.92	3.29	SOS cell division inhibitor
	*umuC*	13.56	5.11	NP	4.46	DNA polymerase V subunit
	*umuD*	14.35	8.31	NP	4.90	DNA polymerase V subunit
	*uvrA*	2.60	2.16	2.47	2.08	Excinuclease ABC subunit A
	*ydjM*	2.30	2.02	1.31	1.12	LexA-regulated gene
	*yebE*	4.84	3.76	4.59	2.37	Putative inner membrane protein
	*yebF*	5.08	2.58	4.89	2.00	Putative secreted Protein
	*yebG*	11.08	15.76	7.74	6.03	DNA damage-inducible protein
Replication	STMUK_0980	2.45	2.33	1.12	3.013	Putative replication regulatory protein
Metabolism	STMUK_2034	2.58	2.03	5.49	2.505	Putative endoprotease
	STMUK_4015	3.00	3.08	2.46	1.47	Putative acetyl esterase
Transport	*ompS*	2.99	2.04	14.14	1.36	Putative porin
	*setB*	4.17	3.01	1.14	2.70	Proton efflux pump
Translation	*asnT*	6.53	6.00	−1.18	−3.06	tRNA-Asn
	*cysT*	2.21	2.62	3.01	1.30	tRNA-Cys
	*glyW*	2.13	2.77	4.37	1.61	tRNA-Gly
	*leuW*	2.67	2.09	3.34	2.33	tRNA-Leu
	*leuZ*	3.40	3.17	1.58	1.51	tRNA-Leu
	*metW*	2.08	2.39	1.22	1.80	tRNA-Met
	*tyrT*	2.26	2.82	−1.27	1.94	tRNA-Tyr
	STMUK_3506	3.75	9.86	5.78	NP	Putative ribonucleoprotein-related protein
Virulence	STMUK_1011	16.94	3.11	13.26	13.73	Attachment/invasion protein
Hypothetical Proteins	STMUK_0985	2.54	4.97	2.98	1.04	Hypothetical protein
	STMUK_0986	4.54	3.45	10.26	1.83	Hypothetical protein
	STMUK_1493	2.50	4.11	3.12	2.788	Putative outer membrane protein
	STMUK_1849	5.81	13.81	4.77	28.65	Hypothetical protein
	STMUK_2239	9.29	21.77	−1.26	15.76	Putative inner membrane protein
	STMUK_2240	15.57	28.49	1.27	59.26	Putative inner membrane protein
	STMUK_2655	4.77	4.67	3.39	1.76	Hypothetical protein
	STMUK_2656	5.04	12.62	12.65	26.71	Hypothetical protein
	*rtcB*	2.61	2.35	1.41	NP	Putative cytoplasmic protein
	*yeeA*	4.79	3.61	3.40	3.42	Putative inner membrane protein
Plasmid	*ccdA*	7.39	4.61	NP	NP	Toxin addiction system: antidote
	*ccdB*	4.32	2.95	NP	NP	Toxin addiction system: toxin
	*rsdB*	7.40	3.17	NP	NP	Resolvase

**^a^** Genes included in column 3 and 4 are >2-fold more highly transcribed with *p* < 0.05 in the UK-1 *dam* mutant strain; **^b^** Grey shading corresponds to genes with higher relative transcription by the UK-1 *dam* mutant against all strains; **^c^** NP: Not Present.

A similar analysis to identify genes stably transcribed during growth in different culture conditions, and more highly transcribed by the *wt* parental UK-1 strain, was performed and identified seven genes, referred to as Gene Set B (Table 2). The average fold change of the seven genes was 4.04 (median 4.04) with a range of 2.53-fold to 6.56-fold in HSLB medium. In LPM medium the average fold change was 3.03 (median 3.03) with a range of 2.00-fold to 5.82-fold. Four functional categories were assigned to the seven genes with the majority involved in metabolism (57%) followed by transport (14%), transcription (14%) and translation (14%). Three genes, *gph*, *rpe*, and *trpS,* fall within the *dam*-containing superoperon which is complexly regulated and contains at least seven genes with generally unrelated function [25,26]. Two genes, *sdaBC*, are involved in serine amino acid metabolism, while *yadI* is a member of the sugar phosphotransferase system. Lastly, *yiaJ* is a repressor of transcription in the IclR family, which is associated with control of some metabolic functions, multi-drug resistance, and quorum sensing [27]. Signal peptide analysis was also performed for Gene Set B and revealed no potential surface expression.

**Table 2:** Gene Set B.

Functional Category	Gene ID	Fold Change of UK-1 *wt* Strain ^a^		Gene Product
		*vs*. UK-1 *dam* Mutant in HSLB Medium	*vs*. UK-1 *dam* Mutant in LPM Medium	
Metabolism	*gph*	4.43	2.41	Phosphoglycolate phosphatase
	*rpe*	6.56	5.82	Ribulose-phosphate 3-epimerase
	*sdaB*	3.56	4.47	L-serine dehydratase/L-threonine deaminase 2
	*yadI*	3.45	2.10	Putative PTS enzyme
Transport	*sdaC*	4.30	4.19	Putative serine transport protein
Transcription	*yiaJ*	2.53	2.11	Transcriptional repressor
Translation	*trpS*	3.43	2.00	Tryptophanyl-tRNA synthetase

^a^ Genes included in the table are >2-fold more highly transcribed with *p* < 0.05 in the UK-1 *wt* strain.

### 2.4. Analysis of Gene Set A in S. Dublin and S. Newport

**Figure 4:**
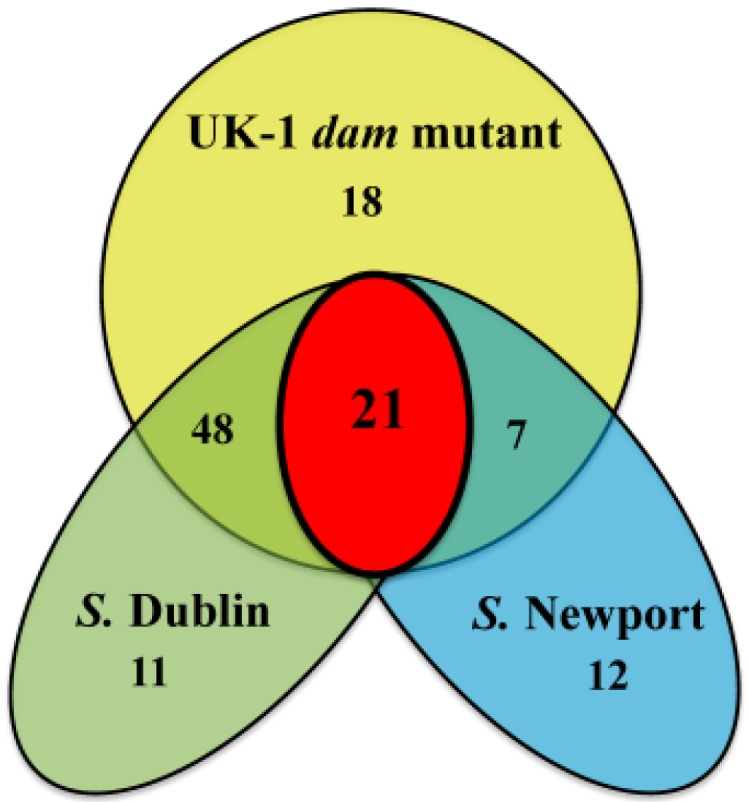
Comparison of Gene Set A transcript levels between the UK-1 *dam* mutant, *S.* Dublin, and *S.* Newport in HSLB medium. Of the 94 genes, 80 are present in the *S.* Dublin genome with 69 genes more highly transcribed by the UK-1 *dam* mutant. Forty genes are present in the *S.* Newport genome with 28 genes more highly transcribed by the UK-1 *dam* mutant. Twenty-one genes are more highly transcribed in the UK-1 *dam* mutant compared to both serotypes examined as well as in both culture conditions when compared to UK-1.

Comparison of Gene Set A transcript levels in the UK-1 *dam* mutant, *S.* Dublin and *S.* Newport in HSLB medium was performed (Table 1, Figure 4 and Supplementary Table 3). Both serotype Dublin and Newport are clinical isolates and were used to determine efficacy of the UK-1 *dam* mutant in cross-protection vaccine studies [17,18]. *S.* Dublin and *S.* Newport had similar sequence identity to the UK-1, (the reference genome) at 99%. Eighty of the 94 genes examined were present in both the UK-1 strain and *S*. Dublin, and of those 80 genes, 69 genes were more highly transcribed by the UK-1 *dam* mutant. In contrast, 40 genes from Gene Set A were present in *S.* Newport with 28 more highly transcribed in the UK-1 *dam* mutant. The lower number of genes present in *S.* Newport reflects the lack of ST64B phage genes in that serotype. Twenty-one of the 94 genes were more highly transcribed by the UK-1 *dam* mutant in comparison to the *wt* parent strain, *S.* Newport and *S.* Dublin in HSLB medium and the *wt* parent UK-1 strain in LPM medium. Twelve of the 21 genes were members of the SOS regulon, which represented the highest numbers of genes in Gene Set A. Other genes included STMUK_3014 of the *std* fimbrial operon, the ST64B bacteriophage gene STMUK_2007, an endoprotease STMUK_2034, a tRNA gene *leuW*, and STMUK_1011, a bacteriophage attachment and invasion protein.

### 2.5. Validation of RNAseq Data with RT-PCR.

**Figure 5:**
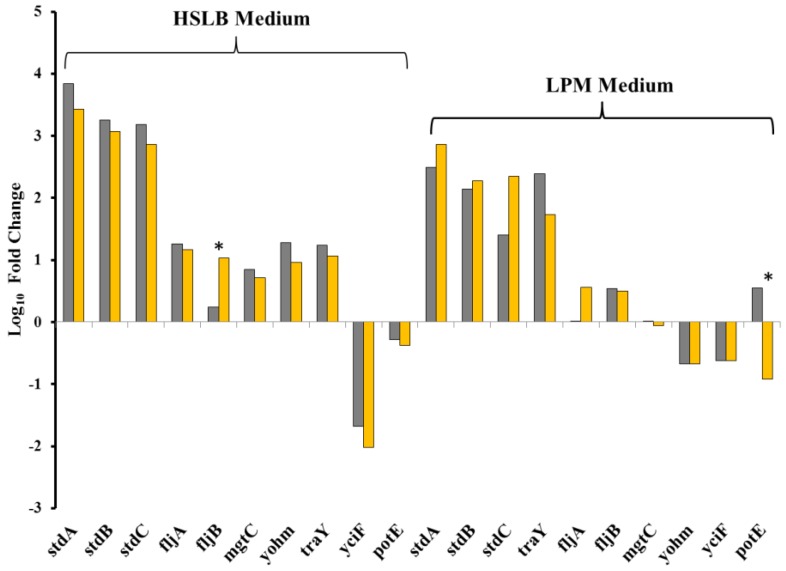
Validation of RNAseq data with RT-PCR in HSLB and LPM medium. Grey bars correspond to RT-PCR values and gold bars correspond to RNAseq data. Positive fold change values indicate the gene was more highly transcribed by the UK-1 *dam* mutant relative to UK-1, the *wt* parent strain. * indicates RT-PCR fold change was not confirmatory of RNAseq data.

Ten genes with a variety of transcriptional statuses in the comparisons performed were used to validate the transcriptome data (Figure 5). According to RNAseq data, eight genes were more highly transcribed by the UK-1 *dam* mutant in HSLB medium, and two genes more highly transcribed by the parental UK-1 strain. These trends were confirmed for 9/10 genes, however *fljB* was not found to be more highly transcribed in the UK-1 *dam* mutant as observed with RNAseq. In LPM medium, four of these genes were more highly transcribed in the UK-1 *dam* mutant, one gene more highly transcribed in UK-1, and five equivalently transcribed by both the UK-1 *dam* mutant and UK-1. The trends of 9/10 genes were confirmed, with *potE* not found to be more highly transcribed by UK-1 as observed with RNAseq. The Spearman rank correlation test found the RNAseq data and the RT-PCR data were equivalent with a value of 0.93 comparing gene transcription in HSLB media and 0.83 comparing gene transcription in LPM media.

**Figure 6:**
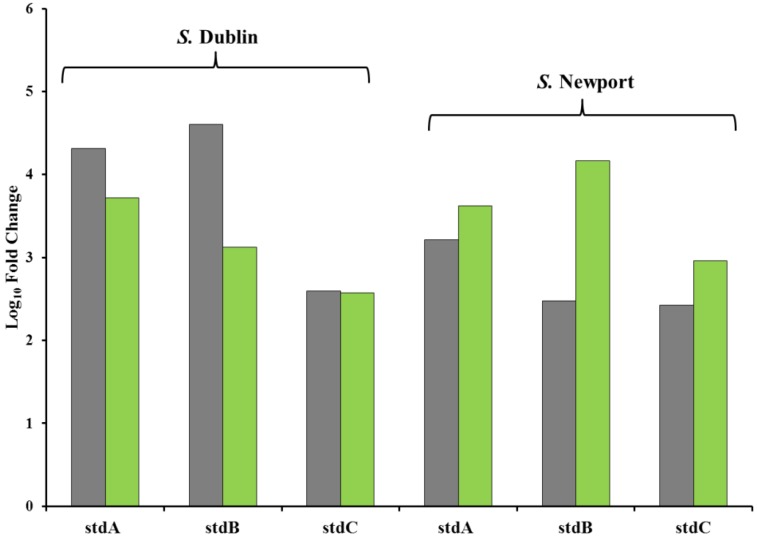
Validation of RNAseq data with RT-PCR comparing the UK-1 *dam* mutant to *S.* Dublin and *S.* Newport grown in HSLB medium. Grey bars correspond to RT-PCR values and green bars correspond to RNAseq values. The UK-1 *dam* mutant was found to more highly transcribe *stdABC* relative to *S.* Dublin and *S.* Newport by both RNAseq and RT-PCR.

RT-PCR was also performed using RNA from *S.* Newport and *S.* Dublin grown in HSLB for the *stdABC* genes, as they were the most highly transcribed by the UK-1 *dam* mutant, to confirm differences observed between the UK-1 *dam* mutant and these strains (Figure 6). The *stdABC* genes were more highly transcribed in the UK-1 *dam* mutant in comparison to *S.* Newport and *S.* Dublin as observed with RNAseq.

## 3. Discussion

The mechanism of protection observed utilizing an *S.* Typhimurium UK-1 *dam* mutant as a live attenuated vaccine is thought to be due to de-repression of gene transcription, with subsequent alteration of protein expression patterns, creating a broad repertoire of antigens that are consistently present in a variety of physiologic conditions [13,14,21]. While de-repression of numerous genes was previously observed, whole genome transcriptional profiling enabled us to detect the full complement of stable genes with higher relative transcription, in the UK-1 *dam* mutant. Our analysis revealed a complement of genes with high relative transcription the UK-1 *dam* mutant compared to the *wt* parent strain that were stably transcribed across host-like microenvironments (Figure 3). Utilizing the intra- and inter-strain comparisons, a subset of 94 genes, referred to as Gene Set A (Figure 3, Table 1), was identified as stably up-regulated by the UK-1 *dam* mutant whereas the *wt* parent strain had only seven genes falling into this subset which were primarily involved in basic cellular processes (Table 2). The genes stably up-regulated by the UK-1 *dam* mutant with potential immunologic significance fall into two categories: genes encoding proteins with predicted surface exposure and genes associated with the bacteriophage ST64B (Table 1). Other genes from this subset are not likely involved in development of an immune response as they are either SOS regulon genes, genes involved in basic cellular processes and/or not predicted to be surface expressed.

The most obvious candidates for classic antigens of all identified genes include proteins encoded by the *std* operon. The *std* fimbrial operon is highly conserved among *Salmonella*, and is present in all three human-adapted *Salmonella* serotypes, Typhi and Paratyphi A and B, where it was first described [28]. Std fimbriae are important in colonization of the bovine intestinal tract, as well as for long-term carriage in the mouse intestine [29,30,31]. While the major fimbrial protein subunit had the highest relative transcription with a 2644-fold and 732-fold in HSLB and LPM media respectively in comparison to the *wt* parent strain, it was excluded from Gene Set A as it was not stably transcribed, with a 3.18-fold increase in HSLB medium. However, high relative transcription in both culture conditions regardless of stability of the transcription supports further evaluation of this gene. The increased transcription of *std* operon genes in the UK-1 *dam* mutant noted here and in previous studies has been shown to be related to an increased protein production. High quantities of Std fimbrial protein, both on the cell surface and in the culture supernatant, have been detected *in vitro* in a different strain of *S.* Typhimurium with the *dam* mutation suggesting protein expression is likely high in the UK-1 *dam* mutant as both strains demonstrate high levels of transcription. [19]. *In vivo*, antibody responses have been detected in mice immunized with wild-type *S.* Typhimurium, as well as to a combination of fimbrial proteins, including StdA, and a decrease in fecal shedding is noted in mice immunized with a fimbrial cocktail [31,32]. The protein encoded by STMUK_3015, which is predicted to be another surface expressed member of the *std* operon, has not been evaluated for antigenicity, but may prove to be a good target for future *in vivo* studies. Comparison of the UK-1 *dam* mutant to two other pathogenic *Salmonella* serotypes identified one gene STMUK_3014, which is not predicted to be surface expressed, with higher relative transcription. Again, the stringency of our stable transcription analysis excluded *stdA*, but the STMUK_3015 was also excluded as it was not present in *S. Newport*. However, the high relative transcription of s*tdA* may still be important in cross-protection as this gene is present in both *S.* Dublin and *S.* Newport for reasons previously mentioned. As immune responses against an Std protein are associated with suppression of *Salmonella* intestinal colonization, our evidence of increased transcription in multiple culture conditions and against multiple serotypes warrants further examination of proteins encoded by this operon [33]. Lastly, in addition to its role as an antigen, it has also been shown that over-expression of Std fimbriae causes enhanced adherence to human colonic epithelial cells in culture, where proximity to resident dendritic cells and macrophages may facilitate antigen delivery at the mucosal surface [34].

Three additional genes encoded proteins with predicted surface expression: STMUK_1011, a bacteriophage attachment and invasion protein, *ydiM*, a Lex-A regulated gene, and STMUK_2003, an ST64B bacteriophage portal gene. Little information is currently available for all three genes as to function or potential immunogenicity of their encoded proteins. Interestingly one gene, STMUK_1011, has higher relative transcription by the UK-1 *dam* mutant compared to the all *wt* strains and which does make it an attractive candidate for further characterization. The significance of these genes is unclear, but the transcriptional differences suggest further investigation is warranted.

We observed bacteriophage ST64B-associated genes to be consistently more highly transcribed in the UK-1 *dam* mutant in comparison to the *wt* parent strain in HSLB and LPM media, as well as *S.* Dublin in HSLB medium. The ST64B bacteriophage is a mosaic structure incorporating different phage types, and is defective in morphogenesis, producing non-infectious tailless particles [35]. Consistent with previous studies, the removal of the Dam enzyme de-represses prophage gene transcription, and in our study the ST64B prophage region represents the largest area of consistent expression in the UK-1 *dam* mutant in both culture conditions [19]. Not only were numerous ST64B genes up-regulated in the UK-1 *dam* mutant in a previous study, but phage DNA and capsid can be found in the supernatant of the UK-1 *dam* mutant bacterial cultures [36]. Unlike the highly conserved *std* operon, ST64B bacteriophage is not highly conserved in *Salmonella* (it was absent from *S.* Newport) and is therefore unlikely to provide cross-protective epitopes [36]. However, ST64B may contribute to development of an immune response through immunomodulatory mechanisms. Use of bacteriophage particles in treatment of bacterial infection in humans has been described as able to modulate host immune responses [37]. Bacteriophages are associated with normalization of inflammatory cytokines, specifically interleukin-6 and tumor necrosis factor-α, in human patients with chronic bacterial infection [38]. There is evidence that the UK-1 *dam* mutant does not induce cytokine production to the same magnitude as wild-type *Salmonella* or other *Salmonella* mutants [39,40]. Bacteriophages are also shown to “prime” macrophages by increasing expression of molecules associated with antigen-presentation [41]. A shift from M2-polarization to a more M1-polarizing milieu has also been shown, indicating the expression of phage proteins may shape a more effective immune response [41]. Therefore, the genes encoding phage proteins could have multiple effects on the host immune response to *Salmonella* proteins and promote more robust and effective protection.

Many other genes have been previously shown to be up-regulated in the UK-1 *dam* mutant, most of which agree with our transcriptome data [19]. However, a few inconsistencies with previous reports were detected. For example, *spvB* was previously shown to be up-regulated in the UK-1 *dam* mutant, however it is more highly transcribed in the *wt* parent strain in our analysis [21]. This likely reflects differences in media composition and incubation time utilized in different studies. In addition, because we have focused on stable genes in different environmental conditions with high relative transcription in the UK-1 *dam* mutant we may have overlooked genes previously studied that did not fit our specific criteria, but which still may be biologically relevant. The wealth of data obtained from this type of sequencing platform calls for targeted analyses to begin to make meaningful conclusions, which does not preclude future analyses of other gene transcription changes.

## 4. Experimental Section

### 4.1. Bacterial Strains

This study utilized *S.* Typhimurium UK-1 strain (ATCC 68169), and a mutant derivative containing a *dam102*: Mu*d*-Cm insertion element (encoding chloramphenicol resistance) disrupting the DNA adenine methyltransferase gene [42]. *S.* Newport and *S.* Dublin were obtained from clinical outbreaks and used in previous experimental animal challenge studies (Table 3) [17,18].

**Table 3:** Strains of *Salmonella enterica* subspecies *enterica* used in this study.

Strain	Clinical Significance	Source	PMID
Typhimurium *dam102*: Mu*d*-Cm MT2313	Avirulent	Heithoff 2001 Deuger 2003	11705984
Typhimurium UK-1	Highly virulent in mice, poultry, pigs, calves and horses	Curtiss 1991 Barrow 2001 Zhang 1999	21622747
Newport 03-721	Wild type isolated from an outbreak of salmonellosis in neonatal dairy calves	Mohler 2002	18329764
Dublin MNO3-9704	Wild type isolated from an outbreak of salmonellosis in neonatal dairy calves	Mohler 2006	16300866

### 4.2. Bacterial Culture Conditions

The UK-1 *dam* mutant and UK-1 were cultured in (i) LB broth containing an additional 300 mM NaCl (HSLB medium), which is known to induce *Salmonella* Pathogenicity Island-1 and stimulate gene transcription similarly to the intestinal lumen; and (ii) a low magnesium and phosphate (LPM) medium containing 5 mM KCl, 7.5 mM (NH_4_)_2_SO_4_, 0.5 mM K_2_SO_4_, 38 mM glycerol (0.3% v/v), 0.1% casamino acids, 8 μM MgCl_2_, 337 μM PO_4_, and 80 mM MES (for titration to pH 5.8) which is known to induce *Salmonella* Pathogenicity Island-2 genes and stimulate gene transcription similarly to the intracellular environment [22,23]. *Salmonella* serotypes Newport and Dublin, were cultured in HSLB medium only. Briefly, one colony of each strain of bacteria was inoculated into LB broth and grown at 37 °C for 16 h at 225 r.p.m. The overnight broth culture was subcultured at a 1:100 dilution into HSLB medium or LPM medium and grown at 37 °C at 225 r.p.m. for 4 h previously determined as the exponential growth phase. Bacteria were centrifuged at 4000 × g for 20 min prior to use in RNA extraction.

### 4.3. Transcriptome Sequencing and Analysis

Total RNA was extracted utilizing the RiboPure Bacteria Kit following manufacturer’s instructions (Ambion, Grand Island, NY). The purity and quality of RNA was analyzed by both the Nanovue Spectrophotometer (GE Healthcare Biosciences, Pittsburgh, PA, USA) and the Agilent 2100 Bioanalyzer (Santa Clara, CA, USA). Three biological replicates from each strain were combined into a single sample for sequence analysis.

Randomly-primed cDNA was synthesized from total RNA and after library preparation, was sequenced using an Illumina HiSeq 2000 (San Diego, CA, USA) sequencing platform. Sequences obtained from the UK-1 *dam* mutant and UK-1 *wt* strain were mapped to the UK-1 reference genome (Genbank accessions CP002614.1 and CP002615.1) using CLC Genomics Workbench (Cambridge, MA, USA). Mapping parameters used in this study were: mismatch cost: 2, insertion cost: 2, and a deletion cost: 3 with both length and similarity fraction of 0.8. After obtaining RPKM values, normalization by quantiles was performed and these values used to determine fold changes in transcription. Statistical significances of the fold changes were assessed using Kal’s Z-test [43]. *S.* Dublin and *S.* Newport were aligned to both the UK-1 reference genome and individual reference genomes for each strain (Genbank accessions CP001144.1 and NC_011080.1 respectively), to ensure comparisons of gene regions with significant identity. Significant differential transcription was considered at a 2-fold or greater change with a *p*-value < 0.05. Transcriptional data for all strains in all conditions tested was deposited in NCBI Small Read Archive (SRP037769).

### 4.4. Real-Time Reverse Transcriptase—PCR (RT-PCR)

Primers (Integrated DNA Technologies, San Diego, CA, USA) were designed to amplify ten genes chosen from the transcriptomic data in order to confirm differences in transcription observed by RNAseq (Table 4). Two housekeeping genes, *gmk* and *rpoD*, were used to normalize the RT-PCR data [44]. cDNA from total RNA used for Illumina HiSeq sequencing was generated using the Superscript III First Strand Synthesis Kit (Life Technologies, Grand Island, NY, USA) per manufacturer instructions. RT-PCR data was analyzed using the ∆∆Ct method to determine transcription differences between strains [45]. Transcription ratios were compared to the RNAseq transcriptomic data, and correlation determined using Spearman’s rank correlation.

**Table 4:** Primers used in RT-PCR.

Gene ID	Forward Sequence 5ʹ-3ʹ	Reverse Sequence 5ʹ-3ʹ	Product Size (bp)
*stdA*	ACCATCACCAACTCACCCTGTGAT	GTGGTTGCATTGGCGGTATTCAGT	104
*stdB*	AGACGTACCTCAGCTCCGCTATTT	GCATTACTGTTCGCAATGCCGCTA	133
*stdC*	AGGACAGGGAAACACTGTTCTGGT	TCCGCTCAGCAGTCAGCTTTCTTT	171
*fljA*	CGTAAATGCGTGTCAGGTTGGTGT	TGATCCTGCTCACCCAGTCAAACA	80
*fljB*	ACCGTTTCACCGCGAACATCAAAG	TCAGTGGTCTGCGCAATGGAGATA	82
*mgtC*	ATTTACTGGCCGCTATGCTGTTGG	TAGTGCGTAATCCCGCCATACGTT	81
*yohM*	AGGTACGGTTAAACAGGCCGTCAT	TTCCACTGATTGTGCGGTGAATGC	124
*yciF*	ATTACGAAATCGCCAGCTACGGCA	TCGTCGAGGGTTTCTTTGAGCAGT	94
*potE*	AATAGCCTTCGTCAGCGGAGGATT	TTTGGTCTGGCGTTTGCACAGATG	145
*traY*	TGTGAGGAGGAGAAACGCAAGAGA	TTCCACTCCGATCTTTAGCCCTGA	104
*gmk*	TTGGCAGGGAGGCGTTT	GCGCGAAGTGCCGTAGTAAT	61
*rpoD*	ACATGGGTATTCAGGTAATGGAAGA	CGGTGCTGGTGGTATTTTCA	74

## 5. Conclusions

In summary, the *Salmonella* Typhimurium the UK-1 *dam* mutant yields higher, yet stable, relative transcription in comparison to the *wt* UK-1 parent strain when grown in biologically relevant culture conditions. A subset of 94 genes was identified where transcription was not responsive to change in environment that was more highly transcribed by the UK-1 *dam* mutant in comparison to UK-1. This finding is in contrast to the UK-1 strain, which stably up-regulated seven genes involved in basic cellular processes. We have provided additional evidence to support the role of *std* fimbrial genes as encoding potential antigens of importance in development of a protective immune response. Most interesting is the possibility of the ST64B bacteriophage gene products contributing to immunomodulation, and perhaps being as important as specific antigens in the protective effect observed using the UK-1 *dam* mutant as attenuated vaccine. It is conceivable that because so many unique transcription differences were observed comparing the UK-1 *dam* mutant to one serotype, that other genes, and their respective proteins, may be involved in protection from disease in a serotype-specific manner. Further examination of the transcriptomes in both culture conditions may also elucidate additional genes potentially contributing to development of protection.

## Supplementary Files

Supplementary File 1
