# Supplementary material for: Transcriptional Profiling of a Cross-Protective Salmonella enterica serovar Typhimurium UK-1 dam Mutant Identifies a Set of Genes More Transcriptionally Active Compared to Wild-Type, and Stably Transcribed across Biologically Relevant Microenvironments
Source: Pathogens. 2014 May 9;3(2):417–36. doi: 10.3390/pathogens3020417 (PMC4213855; doi:10.3390/pathogens3020417)

**Supplementary Figure 1.** Volcano plots of inter-strain transcription comparing the UK-1 *dam* mutant and *wt* parent strain in a) HSLB and b) LMP media. The x-axis is the log_2_ transformed fold change. The y-axis represents the -log_10_ transformed p-value. The vertical red lines denote a 2-fold change in transcription while the horizontal red line represents statistical significance at p<0.05. The genes represented by X’s in the upper right and left areas are considered significant as they represent genes more highly transcribed by either the mutant or parental strain in direct comparison.


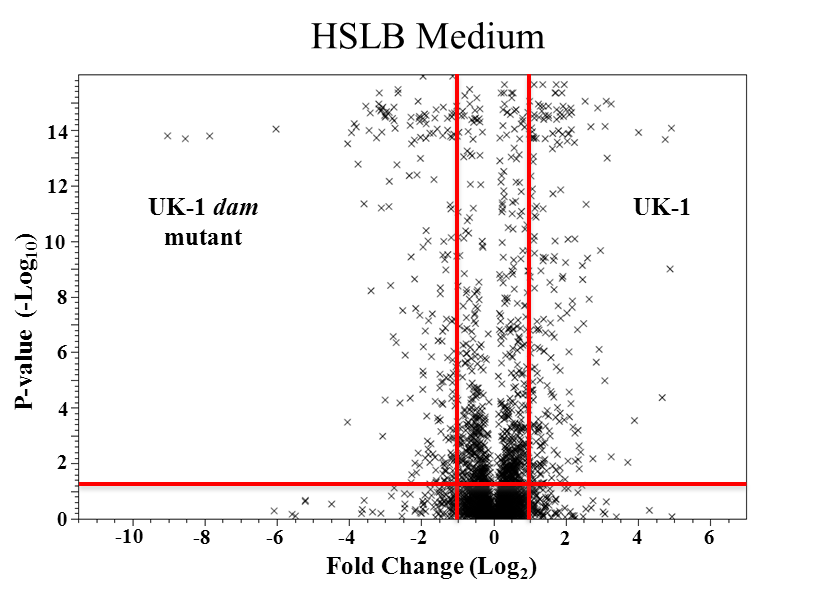

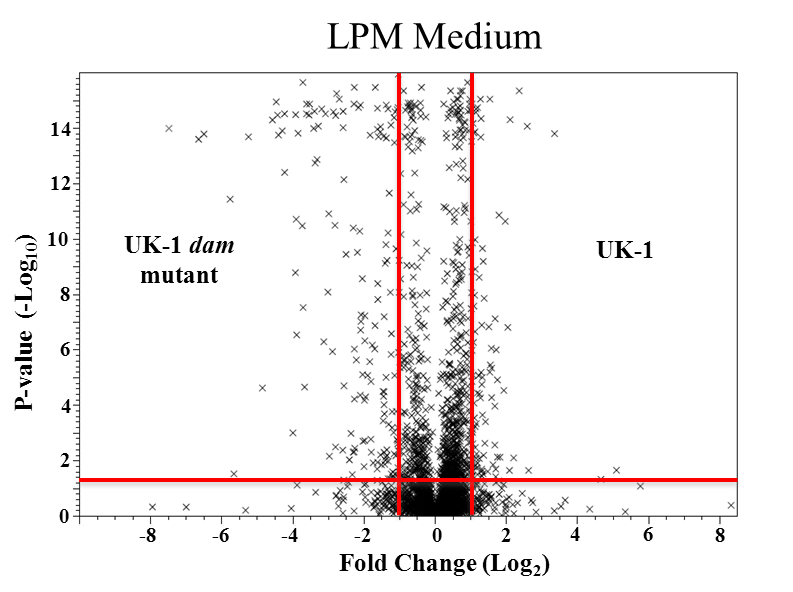

Supplement: Supplementary File 1 [file pathogens-03-00417-s001.zip › Pathogens-52229-Supplementary-final/Supplementary Figure 1.docx]
